# Mobile Apps for the Care Management of Chronic Kidney and End-Stage Renal Diseases: Systematic Search in App Stores and Evaluation

**DOI:** 10.2196/12604

**Published:** 2019-09-04

**Authors:** Abu Bakkar Siddique, Mary Krebs, Sarai Alvarez, Iris Greenspan, Amit Patel, Julianna Kinsolving, Naoru Koizumi

**Affiliations:** 1 Schar School of Policy and Government George Mason University Arlington, VA United States; 2 University of Massachusetts Boston Boston, MA United States

**Keywords:** mobile apps, mhealth, digital health, kidney patient, nutrition tracking, systematic assessment, Mobile App Rating Scale

## Abstract

**Background:**

Numerous free and low-cost mobile apps for the care management of kidney disease have become available in recent years. Although these appear to be promising tools, they have not been evaluated comparatively based on standard mobile app metrics, and thus, limited evidence is available regarding their efficacy. This study systematically cataloged and assessed mobile apps designed to assist medication compliance and nutrition tracking that are useful to the chronic kidney disease (CKD) and the end-stage renal disease (ESRD) patients who are on dialysis.

**Objective:**

The objective of this study was to comprehensively evaluate mobile apps used for medication compliance and nutrition tracking for possible use by CKD and ESRD patients.

**Methods:**

A systematic review framework was applied to the search, screening, and assessment of apps identified and downloaded from the iOS and Android app stores. We selected apps using 13 relevant search terms, narrowed down based on a set of inclusion and exclusion criteria, and then used the Mobile App Rating Scale (MARS), a widely adopted app evaluation tool to assess the effectiveness of apps. The internal consistency and interrater reliability were tested using Cronbach alpha and interclass correlation coefficients (ICCs), respectively.

**Results:**

The MARS total score had excellent internal consistency (Cronbach alpha=.90) and a moderate level of interrater reliability (2-way mixed ICC 0.65). Overall, 11 out of the 12 reviewed apps met the minimum acceptable score of 3.0 in MARS rating. The 3 apps with the highest combined scores were *My Kidneys, My Health Handbook* (MARS=4.68); *My Food Coach* (MARS=4.48); and *National Kidney Foundation Malaysia* (MARS=4.20). The study identified 2 general weaknesses in the existing apps: the apps fell short of accommodating advanced interactive features such as providing motivational feedback and promoting family member and caregiver participations in the app utilization.

**Conclusions:**

The MARS rating system performed well in the app evaluation. The 3 highest ranked apps scored consistently high across the 5 dimensions specified in MARS. These apps were developed in collaboration with reputable organizations and field experts, demonstrating the importance of expert guidance in developing medical apps.

## Introduction

### Background

The prevalence of chronic kidney disease (CKD), particularly end-stage renal disease (ESRD) or the final stage (stage 6) of CKD, has been growing steadily in the United States for the last decade [1]. The increase is primarily attributed to the rising health conditions linked to CKD, such as diabetes, hypertension, and obesity, as well as aging [1]. The most recent statistic reports that CKD affects 14% of the US adult population compared with, for instance, diabetes mellitus (DM), which affects 12% of the population [2]. The treatment costs for CKD, particularly for ESRD, have been a large driver of overall health care spending. The 3 most common comorbidities consisting of CKD, DM, and congestive heart failure together share the highest expenditure for Medicare reimbursement [1]. With the expected further increase in health care costs, improvement in the care for CKD and ESRD patients and the prevention of disease progression have been considered one of the highest priorities in the US health care [3].

Slowing the disease progression requires significant personal involvement for CKD/ESRD patients. The patients face complex recommendations on medication adherence, lifestyle modification, and nutritional adaptation [3]. Meeting specific nutrition guidelines is particularly challenging for ESRD patients on dialysis [4,5]. The burdens of complying with these guidelines are considerable, not only for the patients themselves but also for their families and caregivers (ibid). Previous literature suggests that enhanced knowledge could improve self-management skills in chronic disease [5-7]. However, CKD/ESRD patients are not often satisfied with their actual ability to connect with their health care providers and are mostly unaware of their diagnoses and the implications [8,9].

### Objective

Information technology (IT) tools for monitoring, training, and self-management have been identified as an effective tool to empower patients [4]. The development of IT tools provides patients with access to numerous apps and portals for health information. A vast array of medical reference materials is available to patients through the internet and mobile apps, offering them a better understanding of their diseases and best practices. These apps can not only reduce costs and burdens on others but can also assist in tracking diet and nutrition, recommend healthy foods nearby, supplement medical intervention through drug information, identify pills, check drug interactions, and record personal medication. They can also estimate kidney function, provide diagnostic tests and information on disease signs and symptoms, function as medical calculators, and help manage the progression of CKD. Although these appear to be promising tools, physicians and patients are often overwhelmed with the profusion of these low-cost technologies, which limits their utilization of such innovations [10]. Concurrently, very few apps have a Food and Drug Administration (FDA) clearance or any clinical validation. Thus, not much is known about the effectiveness of these apps, especially those aiming at managing a disease or condition (ibid).

Bailey et al [11] systematically reviewed mobile apps available to patients to support outpatient medication self-management and found that hundreds of apps exist in the marketplace with a variety of quality, content, and functions. They recommended that determining optimal capabilities and clinical benefits as well as evaluating the utility of these existing mobile apps are necessary. Although there are studies that assessed the effectiveness of mobile apps supporting DM [12,13], mental health [14], bipolar disorder [15], suicide prevention [16], and asthma [17], those that support dialysis patients have not yet been assessed. Hence, no evidence is currently available regarding the effectiveness of these mobile apps that solely support dialysis patients who indeed follow more stringent diet than others. To fill this knowledge gap, this study performed a systematic review of existing mobile apps supporting CKD/ESRD patients who are on dialysis.

## Methods

### App Search Strategy

A team of reviewers consisting of 3 undergraduate students, a doctoral candidate, and 2 faculty members downloaded the apps and tested the usability of the apps between August 2016 and September 2017. The systematic review methodological framework was applied to the search, screening, and assessment of health-related mobile apps, except for a few instances where the guidelines are not applicable for app reviews.

For the search, we defined search strings developed specifically for nutrition and medication tracking for CKD and ESRD patients. The strings included “kidney” or “kidney care” or “kidney transplant” or “kidney nutrition” or “renal nutrition tracking” or “dialysis” or “dialysis diet” or “renal diet” or “CKD” or “kidney medication” or “kidney medication tracking” or “kidney water tracking” or “kidney transplant medication.” These search terms were derived through an iterative review process encompassing interactions with the app stores, expert physician inputs, and team consensus over the course of several months. This strategy incorporates the medical phrase synonyms for renal failure, as well as the layperson alternatives to the identified terms, as the affected population of interest may not use the technical terms of the kidney disorder.

During a 1-week window in July 2016, the 13 search terms were used by 3 reviewers to identify publicly accessible apps supportive of nutrition and medication tracking for renal patients. Each of the 3 reviewers utilized different but commonly used devices: (1) an iOS iPhone 5 (Apple Inc), (2) an Android Optimus (Samsung), and (3) a first-generation iPad (Apple Inc). The apps considered were those displayed by the US Google Play Store for Android-based and Apple App Store for iOS-based devices. One member of the team screened the Google Play Store, whereas 2 members screened the Apple App Store to compile cursory descriptions of available apps. The apps were also initially searched through various sites including the FDA medical device website and the *mHealth Database* developed by United States Agency for International Development and African Strategies for Health to verify that both the Google Play Store and Apple App Store were adequate sources for apps and that we do not need to expand our search to other databases [18]. Our final search was performed only on Google Play Store and Apple App Store.

### App Selection Strategy

Apps were selected based on the information included in app name, publisher’s description, and price. Apps were selected for inclusion based on the following criteria: (1) available in English language, (2) free of charge, (3) smartphone app, (4) available for download from the official app stores of Apple or Google, (5) targets patients with kidney disease, and (6) targets patients with renal failure (as intended by the publisher). Duplicate apps extracted on the same device platform were disqualified, whereas different versions of the same app that appeared across platforms were retained for comparison [15]. The remaining apps were then screened for inclusion criteria, and those apps that do not target patients with kidney disease (CKD/ESRD, renal failure, dialysis, etc) were removed.

The team then met to discuss the apps that require additional scrutiny and to jointly determine the final set of apps to be downloaded and installed. This process removed apps with too few features to be considered for an effective care management system. Additional apps were disqualified subsequent to the installation based on several exclusion criteria, as specified in the Results section. Although rankings of apps in stores are in constant flux and updated and rated by app stores and their users [19], the apps selected by our team reflect those most visible to users seeking assistance at the time of selection. This approach is consistent with a representative user experience where the renal patient is subjected to unsystematic availability of apps for support.

### App Data Analysis

This study used a rigorous assessment framework, known as the Mobile App Rating Scale (MARS), developed by researchers at the Queensland University. Broadly speaking, MARS developed by Stoyanov et al [20] offers a promising evaluation scheme for classifying and rating the quality of mobile health apps. MARS is built upon the existing body of scholarship between January 2000 and January 2013 and comprises 4 broad categories of *objective quality* criteria, including app *engagement, functionality, aesthetics,* and *information*, and 1 *subjective* *quality* scale based on the 20 to 23 items of MARS subcriteria. Although *engagement* addressed questions such as “is it fun, interesting, customizable, interactive, well-targeted to the audience?,” *functionality* measured app functioning level, reflecting how easy it is to learn or navigate, flow logic, and gestural design of apps. The score of *aesthetics* assessed the apps’ graphic design, such as overall visual appeal, color scheme, and consistent style. The quality and quantity of information, credibility of the sources of information, evidence, etc, built the score of *information* criterion. The app’s *subjective* *quality* assessed overall satisfaction level and whether the app is worth recommending, stimulates repeat use, etc. MARS has demonstrated excellent internal consistency and interrater reliability (ibid).

To evaluate the final set of apps, 3 reviewers performed scoring for the apps on a 5-point scale, producing a comprehensive final mean score for each app. The internal consistency of the MARS quality subscales and total quality score were calculated using Cronbach alpha. This alpha coefficient indicates the degree (correlations) to which items measuring the same general construct produce similar scores. Interclass correlation coefficients (ICCs) were calculated using 2-way mixed effects for agreement [21]. There were 6 apps that were available on more than 1 device. We used independent scores by each reviewer to calculate ICC. For those 6 apps, reviewers had disagreements in their individual scores. However, reviewers reached to a consensus and produced an agreed-upon score after extensive deliberations for each scale for these 6 apps.

## Results

### Overall Assessment

**Figure 1:**
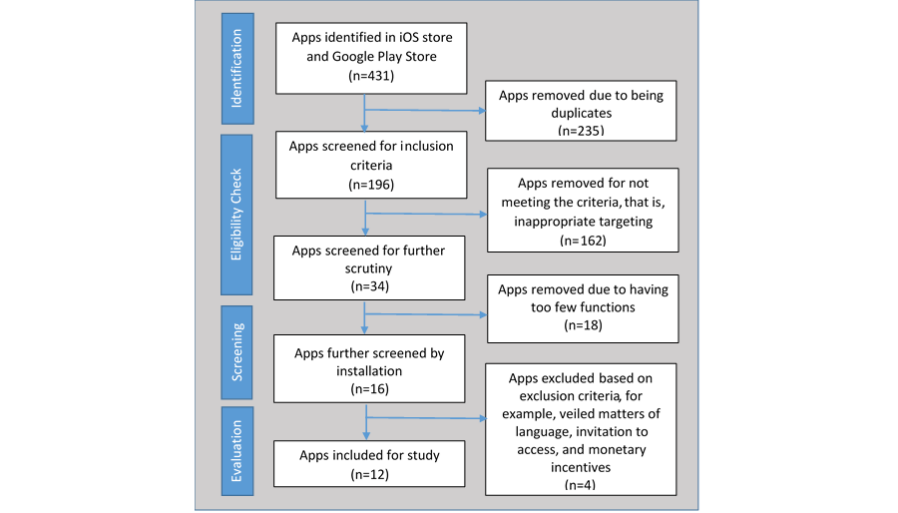
App selection flow diagram.

Figure 1 presents the flow chart of the app selection process. The initial search based on the 13 search terms captured 431 apps. Of these apps, 235 were removed because of being duplicates, which left 196 apps for further evaluation. An additional 162 apps were removed because of not targeting CKD/ESRD patients. Of the remaining 34 apps, a final pool for assessment was determined through discussion and consensus among the team members. This excluded an additional 18 apps that had fairly limited functions. Most of these apps were mere calculators of some sort (estimating calorie, water, and phosphorous intakes), whereas other apps were simple appointment reminders or goal trackers. The remaining 16 apps were subsequently downloaded for comprehensive evaluation. Additional scrutiny of the downloaded apps removed 4 apps because of veiled matters of language, invitation for access, and concealed monetary motivation by publishers (as detailed in Multimedia Appendix 1). Consequently, the final set of 12 apps went through the final evaluation using MARS.

The initial assessment of the 12 apps revealed that their interface designs typically include data entry, goal and reminder settings, and graphing and analytics of achievements. In terms of the functions, these apps typically included calorie intake calculation, goal setting, reminders, access to social networks, and game elements such as rewards or competition among users. The MARS total score had excellent internal consistency (Cronbach alpha=.90) and was highly correlated with the MARS star rating item (#23), r (12)=0.88; *P*<.001. Internal consistencies of the MARS subscales were also very high except for 1 subscale (Cronbach alpha=.40-.83; median .70). Independent ratings on the overall MARS total score of the 6 apps by 2 raters demonstrated moderate level of interrater reliability (2-way mixed ICC 0.65; 95% CI −4.45 to 0.97), and interrater reliabilities of subscales were fair (ICC −0.61 to 0.88; median .48). Detailed item and subscale statistics are presented in Multimedia Appendix 2.

Table 1 presents the mean and median scores of the 12 apps in each of the 5 MARS dimensions. The apps, taken together, scored higher than the minimum acceptable score of 3.0 in all 5 dimensions.

**Table 1:** The Mobile App Rating Scale’s app quality ratings (1-5).

Criteria	Mean (SD)	Median (interquartile range)	Minimum and maximum
Engagement	3.35 (0.78)	3.40 (1.08)	1.6 and 4.3
Functionality	4.29 (0.63)	4.45 (0.85)	2.9 and 5.0
Aesthetics	3.85 (0.63)	3.90 (0.93)	2.7 and 5.0
Information	3.88 (0.84)	4.25 (1.43)	2.5 and 4.8
Subjective quality	3.60 (0.87)	3.60 (0.75)	1.9 and 5.0

The apps scored relatively low in the engagement dimension primarily because of the lack of interactive feature (mean 3.35; median 3.40). The apps scored high in the functionality dimension with the mean and median scores of 4.29 and 4.45, respectively. Here, the scores were consistently high across the subcriteria except for a few apps that scored low in all dimensions.

Table 2 exhibits the ranking of the reviewed apps and their individual comprehensive mean scores. The comprehensive mean scores ranged from as low as 2.98 to as high as 4.68. The median of the 12 mean scores and the interquartile range (IQR) were 3.70 and 0.78, respectively. All apps except for *Phosphorus Tracker* (MARS=2.98) met the minimum acceptable score of 3.0. The 3 apps receiving the highest combined scores were *My Kidneys, My Health Handbook* (MARS=4.68); *My Food Coach* (MARS=4.48); and *National Kidney Foundation Malaysia* (MARS=4.20). *H2O Overload*, the app ranked fourth best (MARS=4.18), was ranked very close to *National Kidney Foundation Malaysia*. *Phosphorus Tracker* and *Wholesome* consistently demonstrated poor scores across most criteria. In between these extremes, *Care After Kidney Transplant*, *CKD Go!*, *AAKP myHealth Nutrition Guide*, *Kidney APPetite*, Oxalator, and *D-Track - Dialysis Tracker* had medium effectiveness.

**Table 2:** Mobile apps and the Mobile App Rating Scale’s comprehensive scores.

Ranking	Apps’ name	Mobile apps rating scale protocol^a^, mean	Subjective quality score^b^	App-specific score^c^ (perceived impact)
1	My Kidneys, My Health handbook	4.68	5.00	4.20
2	My Food Coach	4.48	4.88	4.20
3	National Kidney Foundation Malaysia	4.20	3.67	4.00
4	H2O Overload	4.18	5.00	4.00
5	CKD Go!	4.10	3.38	4.10
6	Care After Kidney Transplant	4.08	3.75	4.70
7	AAKP myHealth Nutrition Guide	3.63	4.00	2.80
8	Kidney APPetite	3.63	3.25	3.40
9	Oxalator	3.58	2.50	1.90
10	D-Track - Dialysis Tracker	3.55	3.50	3.10
11	Wholesome	3.25	3.25	2.00
12	Phosphorus Tracker	2.98	1.88	2.20

^a^The mean of the mobile apps rating scale’s protocol scores for the 4 criteria: (1) engagement, (2) functionality, (3) aesthetics, and (4) information. It includes items 1 to 19.

^b^The subjective quality score includes items 20 to 23.

^c^App-specific score includes the scores for awareness, knowledge, attitudes, intention to change, help seeking, and behavior change.

### Individual App Assessments

**Figure 2:**
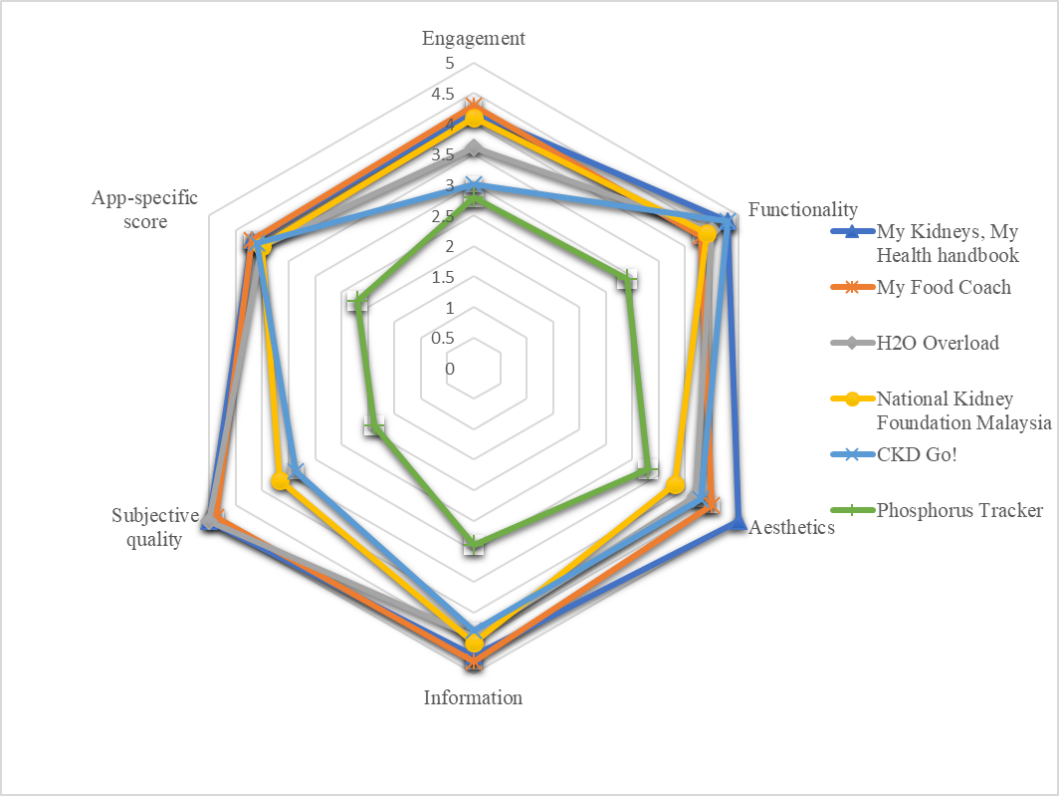
Radar chart of the top 5 and the worst ranked apps.

Figure 2 displays the scores of the top 5 and the worst apps in terms of their assessment criteria. The radar chart demonstrates how these individual apps scored in each criterion such as app engagement, functionality, aesthetics, information, subjective quality, and app-specific score. *Phosphorus Tracker* was ranked far below compared with the top 5 apps across all criteria, but especially in terms of subjective quality and app-specific score. The app was severely penalized for broken links and technical difficulties, that is, crashes and bugs. It is worth noting that the other 5 apps presented in the chart were all developed in collaboration with reputable expert organizations such as the National Kidney Foundation (NKF) in the United States (*My Food Coach*) and in Malaysia (*National Kidney Foundation Malaysia*) as well as the National Health and Medical Research Council (NHMRC) in Australia (the other 3 apps). The high information scores of these apps can be partially attributable to the involvements of these organizations, as credibility and legitimacy of the app source is a subcriterion in the assessment of the MARS information dimension.

The top 2 apps (*My Kidneys, My Health Handbook* and *My Food Coach*) demonstrated similar scores for all criteria, except that *My Kidneys, My Health Handbook* was slightly superior in terms of functionality and aesthetics. The reviewers noted that the app stood out aesthetically with the quality, color coordination, and resolution of the graphics as well as the stylistically consistent interface. The app was also highly interactive and allowed direct access to various support groups. *My Food Coach* received a high score in engagement primarily because of the GPS locator for restaurants and their menus. Similar to *My Kidneys, My Health Handbook*, it was also highly interactive, supporting access to a registered dietitian. *H2O Overload* was considered superior to *My Food Coach* in terms of functionality, whereas it was inferior in all other areas, but especially in terms of engagement and subjective quality. The reviewers noted that the app was memorable for allowing multiple functions such as appointment entry/reminder, medication entry, notepad for questions to physicians, and email to physicians with progression graphs (blood pressure, weight, and fluid intakes). *National Kidney Foundation Malaysia* generally scored similar to *H2O Overload* but stood out for its highly interactive nature, allowing users to set specific and measurable goals and providing feedback. This resulted in its high engagement score. *Care After Kidney Transplant* demonstrated inconsistent quality across criteria: although the app received high scores for functionality, information, and app-specific criteria, engagement and subjective quality criteria were deemed poor. The app excelled in terms of simple and intuitive functionality, links to abundant information, and overall sense of professionalism, but it lacked the customization and interactive features, which affected the engagement score.

## Discussion

### Principal Findings

This study performed a systematic review of the 12 apps that assist CKD and ESRD patients with their care management. To our knowledge, this is the first study that evaluated apps supporting CKD/ESRD care. The evaluation of these apps was performed using MARS. MARS has been successfully applied in recent years to evaluate apps that assist mindfulness development [22], heart failure symptom monitoring and self-management [23], weight management [24], palliative care among pediatric patients [25], epilepsy self-management [26], and drug-drug interaction checking [27]. Our evaluation demonstrated that the overall scores of our apps were comparable with those of apps in the other fields, with the median score of 3.70 (IQR 0.78). All of our apps exceeded the minimum acceptable score of 3.0, except for 1 app (*Phosphorus Tracker*). Overall, the reviewers observed that those apps scoring high (or low) in 1 dimension in the MARS tend to score high (or low) in other dimensions as well. Previous app reviews using MARS note correlations between MARS scores in several dimensions [27]. Such correlations are considered to be particularly evident between the aesthetics and engagement dimensions [28-30]. Although the tendency was also observed in our review, it appeared that the overall similarities in the scores across dimensions were more attributable to the overall professionalism (or lack thereof) of the app developers. The only exception was *Care After Kidney Transplant*, which scored relatively low in the engagement dimension because of the limited customization and interactive features but scored high in all other dimensions.

Most apps had a combination of the functions that support self-management such as appointment/medication reminder and water/weight/phosphorus calculation and monitoring. However, none of these apps incorporated more advanced support functions such as providing motivational feedback to their progress or response to the measured water/weight/phosphorus levels. The relative unavailability of such functions is also noted in previous studies [23,24,26]. Furthermore, the apps reviewed in this study solely focused on CKD/ESRD patients and not their caregivers, and there was no explicit involvement of family members or caregivers in the use of the apps. Apps that promote participation of caregivers/parents are available and are known to be effective in pediatric palliative care [25]. Given the considerable roles played by family members and caregivers in the CKD/ESRD care management, more participatory approach in the design of the apps would be beneficial.

Our findings also indicate that guidelines for app developers are needed. Currently, there are limited resources and information available for the developers to refer to as they develop apps for health services. A large body of the free or low-cost medical mobile apps was made available by unknown publishers without the participation or inputs from reputable domain experts. For instance, 1 app claimed that it could heal kidney problems with sound frequency therapy. In contrast, all high-ranked apps were developed in collaboration with the reputable organizations/field experts such as the NKF in the United States and Malaysia as well as the NHMRC in Australia.

Several observations were made on the app quality and the performance of devices and platforms used to find appropriate apps. Each device (ie, iOS iPhone 5, an Android Optimus smartphone, and a first-generation iPad) identified a varied number of apps, with the first-generation iPad producing the least number of kidney care–related apps and the Android Optimus smartphone producing the largest number of kidney care–related apps. Regarding the platform, reviewers unanimously agreed that the Apple store produced a robust and narrowly refined selection of apps, whereas finding apps in the Google Play store was numerically overwhelming. In particular, irrelevant gaming and entertainment apps were included in the Google Play search. For instance, the app “Crazy XMas Santa Doctor Mania” was included because the description stated, “Be the crazy surgeon doctor to rescue Christmas Santa from… kidney problem…” Thousands of similar apps were captured initially during the Android search. This observation suggests that the Google Play store search-inclusion algorithm could be improved to aid a kidney failure patient in finding an appropriate quality app. In terms of the app quality, MARS scores exhibited no striking differences in terms of quality between apps downloaded from the iOS versus Android app stores.

Finally, there are limited monitoring and regulatory authorities to oversee the fraudulent apps. In the United States, the FDA provides guidance on which mobile apps they regulate and how to regulate. However, the monitoring takes a risk-based approach and is applied only to those apps that meet the regulatory definition of *device*, which operates as an accessary to a regulated medical device. As such, an authority providing guidelines or performing systematic evaluation of apps is warranted.

### Limitations and Future Research

Although this study performed a consistent evaluation of the apps based on the consensus among the team members, an internal critique of the MARS rating scheme offers limitations for consideration. Self-reported limitations by the Queensland developers identify a lack of peer-reviewed literature on which to base the evaluation. There are few other tools to evaluate apps such as App Chronic Disease Checklist and Royal College of Physicians checklist. Cross-checking using these available tools may provide different findings from MARS. Such work could also provide stronger evidence to support CKD/ESRD patients and their physicians.

The interrater reliability of the reviews was fair but differed significantly across subscales. The interrater reliabilities for the engagement and functionality subscales were notably low despite the fact that all the reviewers received a Web-based MARS tool training a priori, followed by a team discussion utilizing a consensus approach to discrepancies in rating. The low interrater reliabilities for engagement and functionality may reflect inherent drawbacks in the MARS instruments, that is, whether one finds the app *interesting* or *engaging* depends heavily on his or her background, whereas individual aptitude for and experience in using apps in general could heavily influence perceived *ease of use and navigation*.

Another limitation is that the reviewers of apps in this study were not real CKD/ESRD patients. Rather, they pretended to be the patients. In addition, the reviewing team was quite small with a smaller number of devices, which may result in biased reviews. Future research should exploit a bigger team, more devices, and clinical trials with actual ESRD patients. A randomized control trial approach may also produce a more reliable result. Finally, as apps are perpetually being developed, so are the apps that support CKD/ESRD patients. A follow-up app search performed in early 2019 revealed that there are a few new apps available in the Apple and Google Play stores. However, these apps are rated relatively low compared with those reviewed in this study, with a low number of downloads. Furthermore, 5 of the 12 apps reviewed in this study appear to be unavailable in the app stores (*National Kidney Foundation Malaysia*, *Care After Kidney Transplant*, *AAKP myHealth Nutrition Guide, Kidney APPetite,* and *Phosphorus Tracker*). Although the 2 apps that scored the highest in this study seem to remain as the leading apps in the field, these findings indicate that evaluation of existing apps should be a continuous effort.

### Conclusions

There has been an explosion of free mobile apps for tracking health in recent years. Although these apps appear to be promising tools, there has been a limited number of studies that systematically evaluate these apps, thereby burdening potential users of these apps with the responsibility to identify the apps that fit to their purposes. For some of these potential users, spending time and other resources to find the best app itself can be a challenging task, not to mention mastering the skills to use these apps effectively. With this in mind, this study conducted an evaluation of apps designed to assist CKD/ESRD patients who are under strict dietary and medication controls and tend to have limited recourses and capacity to explore numerous apps on their own. We used the MARS evaluation tool and identified the top 3 mobile apps supporting CKD/ESRD patients: *My Kidneys, My Health Handbook*; *My Food Coach*; and *National Kidney Foundation Malaysia*.

## Appendix

Multimedia Appendix 1 List of apps excluded from the review.

Multimedia Appendix 2 Interrater reliability and internal consistency of the mobile apps rating scale items.

## Abbreviations

CKD: chronic kidney disease
DM: diabetes mellitus
ESRD: end-stage renal disease
FDA: Food and Drug Administration
ICC: interclass correlation coefficient
IQR: interquartile range
IT: information technology
MARS: Mobile App Rating Scale
NHMRC: National Health and Medical Research Council
NKF: National Kidney Foundation
