# Supplementary material for: Mobile Apps for the Care Management of Chronic Kidney and End-Stage Renal Diseases: Systematic Search in App Stores and Evaluation
Source: JMIR Mhealth Uhealth. 2019 Sep 4;7(9):e12604. doi: 10.2196/12604 (PMC6753688; doi:10.2196/12604)
Supplement: Multimedia Appendix 1 [file mhealth_v7i9e12604_app1.pdf]

Appendix I: List of Apps Excluded from the Review

The following apps were installed and reviewed but subsequently excluded from the final scoring for the given reasons.

- *AJKD (American Journal for Kidney Disease)*: cannot be accessed, must buy subscription, & intended for medical professionals
- *Dialysis of Drugs*: must purchase a subscription
- *Piedmont Kidney Pancreas Transplant*: requires permission, need to enter physician referral code
- *7Med Kidney Care*: technical difficulties - incompatible with iPad (requires iOS 8.0), Android (requires version 4.2 +), and iPhone (cannot create a profile based on city error)
