# Supplementary material for: Mobile Apps for the Care Management of Chronic Kidney and End-Stage Renal Diseases: Systematic Search in App Stores and Evaluation
Source: JMIR Mhealth Uhealth. 2019 Sep 4;7(9):e12604. doi: 10.2196/12604 (PMC6753688; doi:10.2196/12604)
Supplement: Multimedia Appendix 2 [file mhealth_v7i9e12604_app2.pdf]

Appendix II: Interrater reliability and internal consistency of the MARS items

Interrater reliability (based on 2 raters and 6 apps) and internal consistency of the MARS items and subscale scores, and corrected item-total correlations and descriptive statistics of items, based on independent ratings of 12 apps.

Subscale/item	Item-Total Correlation	Mean	SD
**Engagement [Alpha = 0.83, ICC = 0.24, 95% CI: -6.27-.92]**			
1. Entertainment	0.74	3.90	1.07
2. Interest	0.68	3.95	1.15
3. Customisation	0.23	2.60	1.14
4. Interactivity	0.44	2.15	1.09
5. Target group	-0.30	4.37	0.76
**Functionality [Alpha = 0.75, ICC = 0.29, 95% CI: -4.09-.90]**			
6. Performance	0.52	4.15	1.27
7. Ease of use	-0.23	4.35	0.75
8. Navigation	0.37	4.30	0.98
9. Gestural design	-0.28	4.35	0.99
**Aesthetics [Alpha = 0.75, ICC = -.61, 95% CI: -10.46-.78]**			
10. Layout	0.65	4.26	1.05
11. Graphics	0.55	3.84	0.76
12. Visual appeal	0.63	3.84	0.76
**Information [Alpha = 0.40, ICC = 0.84, 95% CI: - -.17-.98]**			
13. Accuracy of app description (in app store)	-0.26	4.58	0.84
14. Goals	0.32	3.46	0.78
15. Quality of information	0.85	4.44	0.62
16. Quantity of information	0.52	4.26	0.93
17. Visual information	0.60	4.25	0.45
18. Credibility	0.72	3.89	1.63
19. Evidence Base*	-	-	-
**App Quality [Alpha = 0.93, ICC = 0.88, 95% CI: -.15-.99]**			
20. Would you recommend this app to people who might benefit from it?	0.88	3.68	1.11
21. How many times do you think you would use this app in the next 12 months if it was relevant to you?	0.73	4.05	0.85
22. Would you pay for this app?	0.86	3.00	1.50
23. What is your overall star rating of the app?	0.84	3.72	0.83
**App Subjective Quality [Alpha = 0.88, ICC = 0.66, 95% CI: -1.44-.95]**			
1. Awareness	0.83	3.74	1.33
2. Knowledge	0.84	4.00	1.37
3. Attitudes	0.57	3.47	0.96
4. Intention to change	0.57	3.58	0.77
5. Help seeking	0.78	3.74	1.37
6. Behavior change	0.51	3.53	0.90
* #19 of MARS score was not used due to missing data			
